# Social predictors of doctoral student mental health and well-being

**DOI:** 10.1371/journal.pone.0274273

**Published:** 2022-09-09

**Authors:** Feng Zhang, Kaylee Litson, David F. Feldon

**Affiliations:** 1 School of International Studies, Binzhou Medical University, Shandong, China; 2 Department of Instructional Technology and Learning Sciences, Utah State University, Logan, Utah, United States of America; University of Oulu, FINLAND

## Abstract

Graduate students’ mental health and well-being is a prominent concern across various disciplines. However, early predictors of mental health and well-being in graduate education, specifically doctoral education, have rarely been studied. The present study evaluated both the underlying latent classification of individuals’ mental well-being and predictors of those classifications. Results estimated two latent classes of students’ mental health and well-being: one class with generally high levels of mental well-being and one with lower levels of mental well-being. Regression analyses showed that mentoring in the second year of doctoral study, certainty of choice in the third year, and both academic development and sense of belonging in the fourth year were positive predictors of membership in the higher mental well-being class. In contrast to some prior studies, demographic variables were not related to the identified well-being classifications. Regression analyses further showed that mental well-being was negatively related to participants’ number of publications and research self-efficacy, indicating a problematic relationship between scholarly productivity and confidence and well-being. These findings may be used to identify and provide targeted support for students who are at-risk for having or developing lower levels of mental well-being in their graduate programs.

## Introduction

Mental health and well-being is an increasingly prominent concern in graduate education [1]. Sixty-eight percent of university presidents acknowledged that, during the COVID-19 pandemic, student mental health ranked among their most pressing issues [2]. Doctoral students are an at-risk population with respect to mental health, illustrated by a study of over two thousand Ph.D. students showing that students in graduate programs are six times as likely as the general population to experience both depression and anxiety [3]. Similarly, recent literature reported high levels of burnout and mental health problems in doctoral biomedical students [4,5]. Further, doctoral students’ research activity may exacerbate depression [6]. The National Academies of Sciences, Engineering, and Medicine [7] recently released results of an 18-month survey on mental health, substance abuse, and well-being in higher education, and urged institutions, faculty, and staff to take action in facilitating and addressing the substantial mental health and well-being concerns of students within higher education.

Graduate students, especially doctoral students, report stressors such as peer pressure [8], high workload [9,10], feelings of uncertainty [11], lack of work-life balance [3], and unproductive mentor-mentee relationships [12]. However, experiences of social support [13,14], academic engagement, financial stability [15,16], appropriate work–life balance [17], and satisfactory supervisory relationships [18] show positive effects on mental well-being either directly or through their mitigation of external stressors. Similarly, a sense of belonging to department, degree program, or laboratory, mitigates negative mental health symptoms [18,19] and correlates positively with academic success and mental well-being [20]. To date, it is unclear when and how such supportive structures impact students’ mental well-being as they navigate through graduate education.

Accordingly, understanding the ways in which aspects of doctoral students’ experiences impact mental well-being is vitally important to assess potential harms or identify positive influences in graduate education processes and structures. Social-environmental factors are highly influential, because socio-psychological well-being hinges on “having supportive and rewarding relationships, contributing to the happiness of others, and being respected by others” (p. 144) [8]. To link the mechanisms and functions of graduate socialization and well-being, this study examines 1) which social-environmental factors impact doctoral student mental health and 2) when during doctoral study do these factors have the largest impact on doctoral student mental health. Specifically we examine the patterns and predictors of Ph.D. student mental well-being in their fifth year of doctoral education by evaluating latent profiles of mental well-being and assessing which factors over time predict students’ membership within those profiles.

### Socialization as a framework for understanding doctoral student experiences

Graduate socialization is defined as “a process of internalizing the expectations, standards, and norms of a given society (discipline), which includes learning the relevant skills, knowledge, habits, attitudes, and values of the group that one is joining” [21]. This process typically unfolds over time as students engage in coursework and supervised research. As students acquire more knowledge and personal autonomy over their research, they gradually internalize a scholarly identity and take on the role of independent scholar. However, individual experiences in these settings vary widely, and maladaptive socialization experiences—such as lack of social support, lack of sense of belonging to departments or labs, and unsatisfactory mentor-mentee relationships—can introduce difficulties or completely derail progress toward degree attainment. Such maladaptive experiences are reported across students from all backgrounds, but those from historically marginalized demographic groups (e.g., women, first-generation college students, people of color) are significantly more likely to experience such difficulties [22–26].

Multiple individuals play important roles in doctoral student socialization. The primary faculty advisor or supervisor (also referred to as “principal investigator” [PI] in some disciplines due to their provision of grant-funded research opportunities and monetary support for the students they supervise) is often considered to have the greatest influence on socialization, due to their direct oversight of students’ scholarly activities and access to resources that shape the development of a research career [27,28]. Similarly, departmental structures and supports can impact the ease and clarity with which students navigate degree requirements [29]. Further, peer interactions both within and outside the immediate research environment can impact day-to-day experiences that influence sense of belonging and emotional well-being [30].

### Predictors of doctoral student mental health

Correlative and predictive studies of graduate and doctoral student mental health have identified multiple relationships with socialization factors. For example, detachment from program activities and negative perceptions of campus atmosphere increase graduate students’ stress levels [31]. Likewise, 44% of doctoral students in another study regarded positive academic socialization as empowering, which correlated positively with research performance and negatively with stress and anxiety [32]. However, doctoral students also identify anxiety related to uncertainty regarding the quality and quantity of their scholarly work as common challenges to their mental health and well-being [33].

Mentorship by supervising faculty likewise plays a major role in graduate, especially doctoral, students’ well-being. High quality mentorship is associated with high levels of mental well-being and life satisfaction, while low quality mentorship can result in stress and depletion [32,34,35]. Positive supervisory faculty relationships are closely associated with increased self-efficacy [36], which, in turn, has a positive relationship with mental well-being [37,38]. Moreover, McAlpine and McKinnon [39] found that when doctoral mentors did not intellectually support students’ work, those students reported elevated levels of both frustration and isolation.

Some studies have suggested that gender and other identity-based demographic characteristics may exacerbate mental health challenges and affect perceived fit in graduate school. Evans et al. [3] reported that transgender students and cis-gendered women were significantly more likely to experience anxiety and depression than cis-gendered men. Likewise, the well-being of women in doctoral programs was hindered by the conflicts they perceived between their own values and priorities and the social structures they depended on [8]. Other external stressors, such as conflicting responsibilities [40], and difficulties in navigating institutional climate [41,42] might deliver more challenges to women compared to men and result in lower level of life satisfaction and mental well-being. International students also encounter elevated stress and mental health challenges due to social isolation and pressures to adapt to new cultural norms [43,44]. Some frequently mentioned stressors that lead to mental health and well-being problems for international students include acculturation difficulties [45–47], lack of social and financial support [48,49], perceived discrimination [50] and marginalization [51,52].

### Current study

Despite high levels of concern, most work previously conducted has evaluated predictors and correlates of mental health and well-being within cross-sectional research. As such, there is little known about the antecedents of mental health and well-being as a function of students’ specific experiences within their doctoral training over time, specifically identifying early predictors that might impact doctoral students’ positive or negative mental well-being. Using the lens of graduate socialization theory in this study [53,54], we use a latent profile approach to identify distinct subpopulations on the basis of mental health and well-being, then examine potential predictors and outcomes of latent profile membership based on primary mechanisms of socialization within doctoral programs.

In the biological sciences, most fifth year Ph.D. students have finished coursework and are working on their dissertations with an eye toward upcoming career opportunities. Consequently, it is a period of multiple potentially stressful transitions and events. For most people, such periods of transition often exacerbate mental health struggles [55]. Thus, the present study has several specific aims: First, based on responses to a survey assessing mental health and well-being, we assess the number of latent profiles that best characterize mental well-being within our national sample. Second, we assess the likelihood of profile membership as a function of demographic variables such as gender, first-generation student status, race/ethnicity, and international student status. Third, we examine the ability of key socialization variables to predict the likelihood of mental health profile membership, including sense of belonging, commitment to degree completion, academic and intellectual development, and mentoring relationships. Lastly, we assess the extent to which mental health profile membership was differentially predicted by academic outcomes, including research skill development, publication output, research self-efficacy, and program attrition.

## Materials and methods

Utah State University IRB has approved our research involving human participants. IRB number is 9317. The written form of consent has obtained.

### Participants and procedures

This study was part of a larger, longitudinal study on Ph.D. student skill development in cellular and molecular biological sciences. Participants were recruited upon entering their doctoral programs in the Fall of 2014 and screened upon entering the study to ensure they met the study criteria of being a doctoral student in the biological sciences in the United States (more information about study criteria can be found in [56]). In total, 336 participants were recruited from 53 institutions across the United States. Of the institutions represented, 42 are classified as R1 (highest research activity), 7 institutions are R2 (higher research activity), and the remaining 4 institutions fall in other Carnegie categories (i.e., Doctoral/Professional Universities).

The present study utilized demographic data collected at the outset of the study, as well as students’ self-reported sense of belonging, certainty of choice, academic development, and mentoring satisfaction collected in the second, third, and fourth year of doctoral study as predictors. The present study further used students’ self-reported mental well-being data collected in students’ fifth year of doctoral study as the primary outcome.

Participants were recruited through one of two methods. First, department chairs and program directors for the 100 largest biological sciences Ph.D. programs across the U.S. were contacted and asked to inform entering doctoral students of the study. Second, emails were sent to listservs relevant to doctoral students in the sciences. To incentivize study participation, students received a $400 annual payment. The full procedure was approved by the Utah State University Institutional Review Board. Participants completed an annual battery of surveys, in addition to providing writing samples and an account of their scholarly productivity each year.

During the first year of the study, students completed a demographic questionnaire that included questions about their race/ethnicity, gender, parents’ education level, and international student status. Each year, participants completed annual surveys about their experiences and productivity. Participants were excluded if they did not respond to at least one item on the mental well-being questionnaire used in the present study, thus a total of *N* = 206 doctoral students were included in the present study. Participants included 40% men, 60% women, and less than 1% non-binary persons; 83% white and Asian students, 17% racially/ethnically minoritized students; 73% continuing-generation students, 27% first-generation students; and 79% domestic students, 21% international students. When assessing the gender and generation status composition across race/ethnicity, the sample included 31 women and 19 men from Black, Latino/a, and Native groups, and 139 women and 84 men from white and Asian groups, 44 women and 30 men who were first-generation college students, and 126 women and 73 men who were continuing-generation college students.

### Measures

#### Demographics

Students indicated their race/ethnicity by selecting one or more of the following: American Indian or Alaska Native; Asian or Asian American; black or African American; Latino/a; Native Hawaiian or other Pacific Islander; white. Students’ responses were aggregated to create a measure of racially/ethnically minoritized status (RMS), where students who selected only a white and/or Asian identity were coded as majority; all other students were coded as RMS (0 = majority; 1 = RMS). To evaluate gender, students self-reported their gender as female, male, and/or other/nonbinary (female = 0; male = 1, other/nonbinary = 2). To evaluate which students were first- compared to continuing-generation college students, students were asked to indicate the highest degree obtained by their parent(s); students who had no parent with a 4-year college degree were coded as first-generation (0 = continuing-generation; 1 = first-generation). Finally, students self-reported whether or not they were an international student (0 = no; 1 = yes).

#### Mental health and well-being

Mental health according to the American Psychological Association, is defined as “a state of mind characterized by emotional well-being, good behavioral adjustment, relative freedom from anxiety and disabling symptoms, and a capacity to establish constructive relationships and cope with the ordinary demands and stresses of life.”**[57]** In the current study, mental well-being was assessed using both a scale of items and a general single-item. The scale of items included 8 items related to mental well-being [58]:

1. I lead a purposeful and meaningful life.
2. My social relationships are supportive and rewarding.
3. I am engaged and interested in my daily activities.
4. I actively contribute to the happiness and well-being of others.
5. I am competent and capable in the activities that are important to me.
6. I am a good person and live a good life.
7. I am optimistic about my future.
8. People respect me.

Items were evaluated on a 5-point Likert scale, from 1 = *Strongly disagree* to 5 = *Strongly agree*. Assuming a single factor structure, the composite reliability was very good (McDonald’s Ω = 0.91). In addition to the mental well-being scale, participants were asked the single question about their mental health “In general, how is your mental health?” with responses ranging from 1 = *Poor* to 5 = *Excellent*. This single item was evaluated in all analyses that included the pre-established measure of mental well-being to evaluate a more robust construction of self-reported mental health alongside mental well-being.

#### Sense of belonging

Sense of belonging to a lab is a subscale of three items from Bollen & Hoyle [59], with an example item of “I see myself as part of the lab/research group community” measured on an 11-point scale with responses ranging from 0 = *Strongly disagree*, *5 = Neutral*, to 10 = *Strongly agree*. Within the present study, this scale showed high reliability, McDonald’s Ω = 0.96.

#### Certainty of choice

Certainty of choice is a subscale of three items from Nora & Cabrera [60], with an example item of “I am certain this institution is the right choice for me” measured on a 3-point Likert scale with responses ranging from 1 = *Strongly disagree*, *2 = Neutral*, to 3 = *Strongly agree*. Within the present study, this scale showed adequate reliability, McDonald’s Ω = 0.86.

#### Academic development and satisfaction

Academic development and satisfaction is a three item subscale from Nora & Cabrera [60], with an example item of “I am satisfied with my academic experience at this institution” measured on a 3-point scale with responses ranging from 1 = *Strongly disagree*, 2 *= Neutral*, to 3 = *Strongly agree*. Within the present study, this scale had adequate reliability, McDonald’s Ω = 0.87.

#### Mentoring relationships

Mentoring relationships were evaluated using 35 items from Graduate Advising Survey for Doctoral Students (GASDS) [61]. The scale utilized four subscales relevant for the doctoral-advisor mentoring relationship: advisor selection criteria (8 items), function of advisor (16 items), satisfaction with advisor (7 items), and time to degree (4 items). Item responses ranged from 1 = *Disagree* to 3 = *Agree*. ***Advisor selection criteria*** represents the match or fit between mentors and mentees, and an example item of this subscale is “My primary advisor is doing research that interests me.” ***Function of advisor*** represents actions an advisor does or does not do that impacts the advisee, and an example item of this subscale is “My primary advisor teaches me strategies for succeeding in my field.” ***Satisfaction with advisor*** exemplifies a mentee’s satisfaction with their primary advisor, and an example item of this subscale is “I currently have the primary advisor I want.” ***Time to degree*** is representative of the support structures implemented to help students progress toward their degree, with an example item being “How helpful has your primary advisor been to you in terms of progressing toward the completion of your degree?” Item subscales (average of each subscale) were evaluated in analyses.

#### Research skills

Year 5 research skills were measured by doctoral students’ sole-authored research paper that was submitted in their fifth year of doctoral study, and were not to have been edited or contributed to by others. Two independent reviewers rated each document on 12 research skills on a scale from 0 to 3.25 according to rubrics [56,62,63]. Interrater reliability as measured by intraclass correlations was good, 0.818 to 0.969. Skills included: introducing/setting the study in context (INT); appropriately integrating primary literature (LIT); establishing testable hypotheses (HYP); using appropriate experimental controls and replication (CTR); experimental design (EXP); selecting data for analysis (SEL); data analysis (ANA); presenting results (PRE); basing conclusions on results (CON); identifying alternative explanations of findings (ALT); identifying limitations of the study (LIM); discussing implications of the findings (IMP) [56]. Skills were evaluated as a composite score in the present study. More information about the measure of research skills are provided in detail in [56].

#### Publication output

A survey on the publication details of the participants was annually administered from year one to year six (2014–2020). Publications were assessed by whether a respondent has a publication or not in a given year (binary variable: 0 for no, and 1 for yes); the number of publications a respondent had each year; total number of authors for each publication; author number of the respondent on each publication (first author, second author, etc.); the titles and journal names of each publication, and the journal impact factor by year for each publication. The publications were then cross-checked with Web of Science and additional sources such as Google Scholar after data collection (see Roksa et al. [64] for more details). Publications within and up to year 5 were included in the present analyses.

#### Self-efficacy

Year 5 research self-efficacy was evaluated using the Research Experience Self-Rating Survey [65]. Questions from this survey asked students to self-rate their ability to perform ten specific research tasks on a 5-point Likert scale (1 = not at all to 5 = a great deal). Items include the ability to complete skills necessary for producing research publications, such as “To what extent do you feel you can understand contemporary concepts in your field?” and “To what extent do you feel you can statistically analyze data?”

### Statistical analysis approach

The primary focus of the study was to examine correlates and predictors of mental health and well-being. We used a person-centered approach to first evaluate mental well-being in order to understand the extent to which we could classify individuals into groups with varying and potentially different responses to the mental well-being questions. Therefore, we first used latent profile analysis to identify latent subgroups of mental well-being and mental health. Latent profile analysis (LPA) is a person-centered quantitative approach used to identify unmeasured subgroups of participant responses to continuous items. Participants were then assigned to a profile with the highest likelihood of resembling their responses to the items, allowing for interpreting results in terms of categorical grouping differences.

After identifying and describing the number of latent profiles, we assigned individuals to profiles and evaluated predictors of the profiles using logistic regression analysis. Specifically, we ran nine logistic regression models to examine predictors of the latent profiles of mental health profiles. Model 1 evaluated static predictors of mental well-being classifications, such as gender, first-generation student status, racially minoritized student status, and international student status. Models 2 through 8 evaluated longitudinal predictors of mental well-being classifications, with Model 2 evaluating the doctoral students’ certainty of choice across years as predictors of mental well-being classifications, Model 3 evaluating Academic Development, Model 4 evaluating Sense of Belonging to a lab, Model 5 evaluating Advisor Selection Criteria, Model 6 evaluating Satisfaction with Advisor, Model 7 evaluating Function of Advisor, and Model 8 evaluating time to degree. Models 9, 10, and 11 respectively evaluated within time predictors of mental health and well-being, including number of publications, research self-efficacy, and research skills. For models that used repeated measures, predictors were evaluated as discrete variables (e.g., Year 2 Certainty of Choice was one variable and Year 3 Certainty of Choice was a separate variable) and were allowed to correlate across all time points. All analyses were conducted in M*plus* version 8.4 [66]. Missing data was handled using full information maximum likelihood with robust standard errors and Montecarlo integration in M*plus*.

## Results

The first step in our analyses entailed evaluation of statistical latent profile models, comparing alternatives that estimated from one to four classes. Next, logistic regression analyses were used to estimate the likelihood of belonging to one of the identified latent profiles as a function of independent variables of interest.

### Identifying profiles of doctoral student mental health

Table 1 shows the model fit indices across these four models, and we evaluated these fit indices in combination with theoretical interpretability of the classes. When evaluating model fit, the Vuong-Lo-Mendell-Rubin (VLMR) likelihood ratio test was used to evaluate the best-fitting model, likewise indicating that the 2-class model was better fitting than the one-class model (VLMR = -2399.49, *p* = .0014), but showing that the three-class model did not fit better than the two-class model. Additionally, the Bayesian Information Criteria (BIC) values continually decreased as more classes were added though the values flattened and created an “elbow” at the 2-class model. Entropy and classification indices were then evaluated, where values closer to 1.0 for both indicate greater accuracy of classification. Entropy was the highest for the two-class model at .95 and classification probabilities for classes 1 and 2 respectively were .97 and .99, indicating very good classification rates of individuals to latent classes. Thus, the two-class model was selected as the best-fitting model and was used in further analysis.

**Table 1:** Fit indices for models with 1, 2, 3, and 4 classes.

Model	Loglikelihood	Number of free parameters	BIC	VLMR	p value	Entropy
1-class	-2399.491	18	4894.885	NA	NA	NA
2-class	-2085.435	28	4320.051	-2399.491	.0014	0.956
3-class	-1973.405	38	4149.268	-2085.435	.2053	0.895
4-class	-1903.474	48	4062.686	-1973.405	.3572	0.909

**Fig 1:**
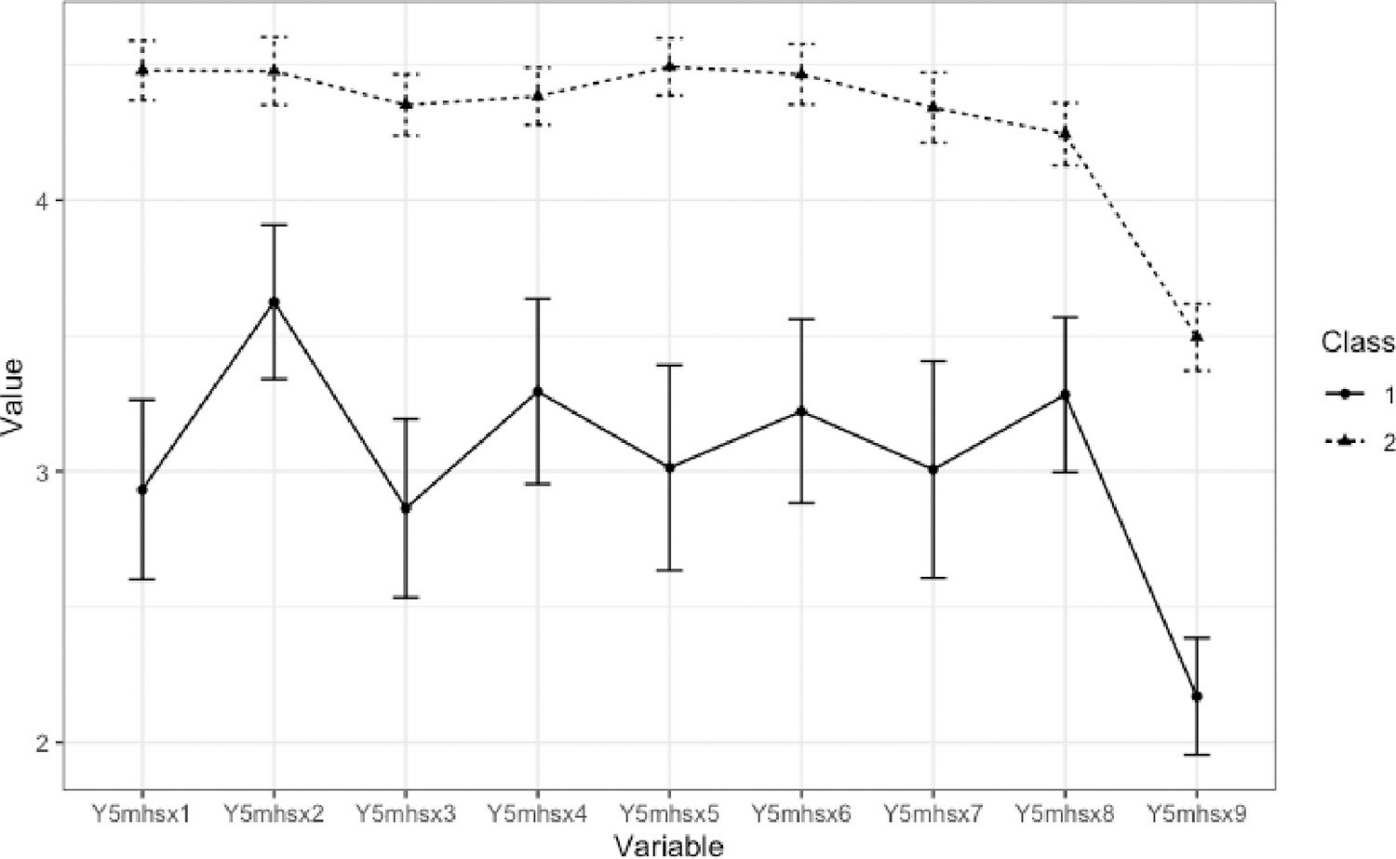
Means across profiles of the 2-class latent profile analysis. Items correspond to the order of items in the mental well-being scale in the methods section.

The two latent classes that emerged included a class with lower and more variable levels of well-being and a class with higher and more stable levels of well-being (see Fig 1). Specifically, all items showed higher mean values in the “higher well-being” profile (*n* = 160) compared to the “lower well-being” profile (*n* = 46). Notably, there is less variability and very high means in the high well-being class, indicating that most students in this class selected maximum values across mental well-being questions. Conversely, the lower mental well-being class showed greater variability in their average scores across items. We additionally evaluated latent profiles examining only the 8 items from mental well-being subscale [55], dropping the self-report mental health item. Results showed essentially the same two profiles. Participant classifications obtained from the 8-item LPA and the reported 9-item LPA were correlated at *r* = .989.

### Predicting doctoral student mental health

Logistic regression analyses were used to assess socialization, skill, and demographic predictors of profile classification. Results were evaluated by examining the 95% confidence interval of the odds ratio (OR). Confidence intervals containing 1.0 indicated that the predictor did not differentially predict classification, while confidence intervals that did not contain 1.0 did differentially predict classification. Results (Table 2) showed no differences in profile classification across gender in reference of men vs other genders (OR = 1.05, 95% CI [0.53, 2.07]), first-generation student status (OR = 0.87, 95% CI [0.45, 1.65]), underrepresented minority status (OR = 1.58, 95% CI [0.68, 3.68]), and international student status (OR = 1.01, 95% CI [0.48, 2.09]). Thus, no differences in the likelihood of being included within either of the mental well-being profiles were associated with demographic characteristics.

**Table 2:** Demographic, academic development, and socialization predictors of year 5 mental well-being.

	Odds Ratio	95% Confidence Interval		
		Lower Limit		Upper Limit
Model 1: Demographic variables predicting mental health and well-being				
Men	1.00	0.51	1.96	
First-generation college student	1.15	0.60	2.21	
Racially minoritized student	0.63	0.27	1.46	
International student	1.00	0.48	2.09	
Model 2: Certainty of Choice predicting mental health and well-being				
Year 2 Certainty of Choice	1.82	0.85	3.85	
Year 3 Certainty of Choice	2.56*	**1.18**	**5.56**	
Year 4 Certainty of Choice	0.86	0.68	1.09	
Model 3: Academic Development predicting mental health and well-being				
Year 2 Academic Development	0.70	0.29	1.69	
Year 3 Academic Development	2.50	0.89	7.14	
Year 4 Academic Development	3.13*	**1.20**	**8.33**	
Model 4: Sense of Belonging predicting mental health and well-being				
Year 2 Sense of Belonging	1.25	0.98	1.61	
Year 3 Sense of Belonging	1.15	0.91	1.47	
Year 4 Sense of Belonging	1.32*	**1.01**	**1.69**	
Model 5: Advisor selection criteria predicting mental health and well-being				
Year 2 Advisor selection criteria	1.69	0.47	5.88	
Year 3 Advisor selection criteria	1.33	0.27	6.25	
Year 4 Advisor selection criteria	1.72	0.52	5.56	
Model 6: Satisfaction with advisor predicting mental health and well-being				
Year 2 Satisfaction with advisor	4.35*	**1.18**	**16.67**	
Year 3 Satisfaction with advisor	1.15	0.29	4.55	
Year 4 Satisfaction with advisor	2.08	0.65	6.67	
Model 7: Advisor function predicting mental health and well-being				
Year 2 Advisor function	1.37	0.49	3.85	
Year 3 Advisor function	3.33	0.71	16.67	
Year 4 Advisor function	1.82	0.46	7.14	
Model 8: Time to degree predicting mental health and well-being				
Year 2 Time to degree	1.96	0.75	5.00	
Year 3 Time to degree	0.99	0.32	3.03	
Year 4 Time to degree	2.38	0.72	7.69	

NOTE:

* = 95% CI does not contain 1.0. Results should be interpreted as follows: e.g., As certainty of choice in year 3 increases, the odds of being in the higher mental well-being profile are 2.56 that of being in the low mental well-being class.

Logistic regression analysis of socialization variables across years yielded many effects (Table 2) that were determined interpretable. To better interpret values, results were all divided by 1 and interpreted accordingly. Certainty of choice in year 3 predicted differences in mental well-being profile classification (OR = 2.56, 95% CI [1.18, 5.59]), indicating that the odds of being in the higher well-being profile was 2.56 times that of being in the lower well-being profile. In year 4, both academic development (OR = 3.13, 95% CI [1.20, 8.20]) and sense of belonging (OR = 1.32, 95% CI [1.01, 1.69]) predicted differences in mental well-being profile classification. One facet of mentorship, satisfaction with advisor, in year 2 was the strongest predictor at the mental well-being classifications (OR = 4.35, 95% CI [1.18, 16.67]).

Logistic regression analysis to evaluate the association between doctoral students’ mental health membership and the number of peer-review publications in year five yielded significant results (Table 3). The number of peer-review publications in year five was related to different mental well-being profile classifications (OR = 0. 61, 95% CI 0.40, 0.91]), indicating a negative association between higher mental well-being and the number of publications in the same year, and the odds for participants with more publications of being in the higher well-being profile was 0.61 times that of being in the lower well-being profile; alternatively, this can be interpreted as the odds for participants with more publications of being in the lower well-being profiles was 1.64 (1/0.61) times that of being in the higher well-being profile. When assessing the research self-efficacy in year five, results likewise showed negative relationships between research self-efficacy and mental well-being (OR = 0.23, 95% CI 0.14, 0.39]), and the odds of being in the higher well-being profile was 0.23 times that of being in the lower well-being profile; in other words, the odds of being in the lower well-being profile was 4.35 (1/0.23) times that of being in the higher well-being profile. Logistic regression analysis results showed that demonstrated research skills in year five did not differentially predict classification in doctoral students’ mental health and well-being.

**Table 3:** Student outcomes and year 5 mental well-being.

Models	Odds Ratio	95% Confidence Interval	
		Lower Limit	Upper Limit
Model 9: Number of Publication predicting mental health and well-being			
Y5-Number of Publication	**0.61** *	**0.40**	**0.91**
Model 10: Self-efficacy predicting mental health and well-being			
Y5-Research Self-Efficacy	**0.23** *	**0.14**	**0.39**
Model 11: Research Skills predicting mental health and well-being			
Y5-Research Skills	1.42	0.65	3.07

NOTE:

* = 95% CI does not contain 1.0.

## Discussion and conclusions

Results identified two stable profiles of doctoral student mental health and well-being, one that reflected consistently positive responses to each item included in the Diener et al. [58] scale (item means from 3.5–4.5 out of 5) as well as a general self-report item of mental health, and one that reflected lower and more variable item responses (item means from 2.2–3.6 out of 5). The odds of belonging to either profile did not differ significantly by doctoral students’ demographic characteristics. These findings are contrary to prior studies which have reported gender differences of experiencing anxiety and depression [3] or mental conflicts in fulfilling multiple roles to maintain life balance [8,67]. Unlike much of the prior research reported on graduate student mental health and well-being, our sample is derived from doctoral students primarily at high research activity (R1) institutions as well as students only from one field of research: lab-based biological sciences. As such, students within this sample may differ from the general doctoral student population. Consequently, finding no demographic differences in mental health and well-being among bioscience doctoral students in this study implies that students have similar levels of mental health and well-being regardless of gender, race, and ethnicity within this population. It is also possible that the present sample size was too small to detect minute differences in mental health and well-being across demographic groups. Future research should take these interpretations and limitations into consideration moving forward.

In contrast, the socialization variables certainty of choice, academic development, sense of belonging, and satisfaction with advisor predicted significant differences in the likelihood of belonging to one latent profile over the other in earlier years of doctoral study. In each case, more favorable scores significantly predicted membership in the latent profile with higher scores on the mental health and well-being scale. Notably, satisfaction with a primary advisor (i.e., dissertation chair, research supervisor) was a significant, positive predictor of positive mental well-being in early years of doctoral education. These results align with prior research that positive, high quality mentoring contributes to positive mental well-being [34]. Further, the satisfaction with advisor subscale is designed to represent actions that the mentor does to help and encourage their student’s success. Results thus show that students who are satisfied with the mentorship they receive in year two are more likely to have greater levels of positive mental well-being, supporting, highlighting the importance of positive, high-quality mentoring experiences between Ph.D. students and their primary advisor as a preventative measure for negative mental health and well-being. Future work on mentorship networks (e.g., receiving mentorship from postdocs, peers, dissertation committee members, etc. in addition to mentorship from the primary advisor) is needed to more fully understand the extent to which mentorship impacts student mental health and well-being.

Of note is that these data were collected longitudinally rather than at a single point in time, which allowed us to assess early predictors of positive and negative mental health and well-being. Specifically, mental health and well-being were evaluated in students’ fifth year of doctoral study, which is a transient time when doctoral students begin their transition to professional careers [12]. Predictors of mental health and well-being, however, were evaluated as early as the second year of doctoral study and were specifically chosen because they are relevant to socialization theory and have the potential to act as intervening variables. For example, the finding that students with positive mentoring relationships in year two were 4.4 times as likely to belong to the high mental health and well-being profile in year five suggests that it may be possible for administrators, student support services, department heads, and other mentors to provide additional support to students who are unsatisfied with their mentor as early as year two. Such intervention may reduce these students’ chances of having negative mental well-being in later years of their graduate education. Further, the finding that participants with lower certainty about their choice to pursue their Ph.D. in year three were 2.6 times as likely to belong to the lower mental health and well-being profile the following year indicates a potentially important risk factor. These factors could potentially be assessed to provide targeted support for students who are more at-risk for having or developing lower levels of mental well-being.

While we are hesitant to claim these relationships as causal, there is one early, strong predictor of student mental well-being in year five; that is satisfaction with the primary advisor in year two. Administrators of doctoral programs may use these findings to implement simple program evaluation assessments to understand student-advisor interactions in the early years of doctoral education to determine whether students are satisfied with their mentorship, to what extent students require additional mentorship or support, and/or to help mentors meet the needs of individual students with the intention to improve (or at least not impair) student mental well-being as students progress through their doctoral programs. Asking questions about students’ mentorship experiences as part of a routine assessment will 1) remove the onus from the student, 2) can help administrators identify students who may be most at risk for reporting negative mental well-being, 3) help mentors identify what their students need since this is not always clear nor communicated, and 4) can provide students with ways to redress mentorship issues, find additional mentorship support, or potentially change mentors. Such intervention programs addressing graduate student and postgraduate mental health have begun to emerge (e.g., [68,69]), and results from studies such as the one presented here show important, early socialization predictors of mental well-being that can and should be addressed at a systematic level to better support doctoral student mental well-being. Intervention programs for mentorship are beyond the scope of this study but may be beneficial for addressing student concerns when unsatisfactory mentorship emerges. Practically speaking, attending to the ways that social-psychological factors influence the well-being of STEM doctoral students points to important ways that faculty PIs and institutional leaders can fine-tune their policy making and (re)shape the values permeating their lab environments, student productivity, and how these features intersect with student mental health and well-being.

Unexpectedly, we found that membership in the more positive mental health and well-being profile was less likely for Ph.D. students who were highly productive in terms of scholarly productivity and for those who were highly confident in their research skills. While it might be assumed that individuals with better mental health are more resilient, confident, and productive, this assumption is not supported by the present data. Thus, it is possible that the pressures associated with sustaining high levels of productivity ultimately erode students’ well-being, even when they are successful in meeting expectations, and perhaps requires a shift in the ideology surrounding academic productivity and positive work environments. Accordingly, faculty mentors and administrators should not assume that simply because a student is demonstrating success in commonly valued metrics such as publication rate that the individual is thriving. Indeed, it may be that an important but neglected aspect of effective mentoring is to guide students to focus their productivity on fewer publications of greater impact. If consistent messaging to students about success as a researcher focuses predominantly on the quantity of publications, it would further explain the finding that students highly confident in their research abilities exhibit lower levels of well-being overall. While the ever-increasing pressure to publish is a normative element of the academy, it may be that endorsing or permitting it to increase unchecked will undermine the scientific enterprise in the long run by harming the very workforce that doctoral education seeks to train as the next generation of scientists.

While the association of these variables with the differential likelihood of being in one of the two latent profiles identified cannot be interpreted as causal in the current study, it can inform the direction of future research intended to foster effective interventions. Future research should ascertain to what extent the findings presented are unidirectional or reciprocal over the course of graduate education. It is additionally important for future research to examine potential differences among mental health and mental well-being, which were not differentiated nor should be interpreted separately in the present study. In addition, it will be important for future research to investigate different possible interventions targeting sense of belonging, mentoring relationships, and perceptions of academic development—constructs central to socialization—as a means of supporting doctoral students’ mental health and well-being. Findings from such work could provide evidence about the function of mental well-being as it manifests during graduate education and identify social factors that can mitigate negative mental well-being and promote positive mental well-being.
